# *C. elegans* episodic swimming is driven by multifractal kinetics

**DOI:** 10.1038/s41598-020-70319-0

**Published:** 2020-09-08

**Authors:** Yusaku Ikeda, Peter Jurica, Hiroshi Kimura, Hiroaki Takagi, Zbigniew R. Struzik, Ken Kiyono, Yukinobu Arata, Yasushi Sako

**Affiliations:** 1 Cellular Informatics Laboratory, RIKEN, 2-1 Hirosawa, Wako, Saitama 351-0198 Japan; 2 Department of Mechanical Engineering, School of Engineering, Tokai University, 4-1-1 Kitakaname, Hiratsuka, Kanagawa 259-1292 Japan; 3 Department of Physics, Nara Medical University, 840 Shijocho, Kashihara, Nara 634-8521 Japan; 4 Graduate School of Education, University of Tokyo, 7-3-1 Hongo, Bunkyo-ku, Tokyo, 113-0033 Japan; 5 Advanced Center for Computing and Communication, RIKEN, 2-1 Hirosawa, Wako, Saitama 351-0198 Japan; 6 Faculty of Physics, University of Warsaw, Pasteur 5, 02-093 Warsaw, Poland; 7 Graduate School of Engineering Science, Osaka University, 1-3 Machikaneyama-cho, Toyonaka, Osaka 560-8531 Japan

**Keywords:** Computational biology and bioinformatics, Genetics, Physiology, Systems biology

## Abstract

Fractal scaling is a common property of temporal change in various modes of animal behavior. The molecular mechanisms of fractal scaling in animal behaviors remain largely unexplored. The nematode *C. elegans* alternates between swimming and resting states in a liquid solution. Here, we report that *C. elegans* episodic swimming is characterized by scale-free kinetics with long-range temporal correlation and local temporal clusterization, namely consistent with multifractal kinetics. Residence times in actively-moving and inactive states were distributed in a power law-based scale-free manner. Multifractal analysis showed that temporal correlation and temporal clusterization were distinct between the actively-moving state and the inactive state. These results indicate that *C. elegans* episodic swimming is driven by transition between two behavioral states, in which each of two transition kinetics follows distinct multifractal kinetics. We found that a conserved behavioral modulator, cyclic GMP dependent kinase (PKG) may regulate the multifractal kinetics underlying an animal behavior. Our combinatorial analysis approach involving molecular genetics and kinetics provides a platform for the molecular dissection of the fractal nature of physiological and behavioral phenomena.

## Introduction

Animal behaviors are organized over a broad range of time scales, ranging from seconds to years, including expansive timescales over lifespan phases, such as infant, juvenile, adult, and elderly phases. Among the temporally organized animal behaviors are rhythmic behaviors characterized by their frequencies. Molecular regulators e.g. for daily rhythmic and oscillatory changes of animal behaviors i.e. for circadian rhythm have been identified and are shown to be regulated by a feedback control^1,2^. Contrastingly, many arrhythmic changes of behavioral and physiological activities in a great variety of animal species, including humans are reported to show self-similar and scale-free structures, which is an indicative for fractal scaling^3–5^ (Fig. S1 and Supplemental Information). Fractal geometry provides a mathematical framework for characterizing scale-free and self-similar patterns^3–5^. Fractal temporal patterns of behavior and physiology have been reported in the locomotion of birds, mosquito larvae, and flies^6–8^, crawling of cultured *C. elegans* worms^9^, clicking sounds produced by feeding sea horses^10^, and swimming of zooplankton^11,12^. In humans, temporal fractal patterns have been observed in wrist movements during habitual sleep/wake cycles^13^, gait^14,15^, heartbeats^16,17^, and brain activity^18,19^. Altered fractal patterns of activity have been associated with pathological conditions and aging^13–15,17,20,21^. Fractal properties of natural phenomena, including physiological and behavioral activities, are not a consequence of the particular biological significance of such activities, but rather due to a common fundamental principle of biochemical reactions in neuromuscular networks. To bridge the gap between macroscopic fractal animal activities and microscopic biochemical reactions in the neuromuscular networks of animals, it is effective to combine a kinetic modeling of animal activities based on fractal scaling with genetic analyses of animal behavior. Such a combinatorial approach remains to be explored.

Owing to its simple anatomy and the availability of a range of genetic tools, *C. elegans* is a powerful model organism for the study of the molecular bases of behavior. In a liquid solution, *C. elegans* worms alternate between swimming and resting states on a minute to hour time scale^22, 23^. In the swimming state, they alternate between continuously beating their bodies and resting with or without food^22–24^. In the resting state, they maintain a characteristic sharply-bent posture^22^. Episodic swimming is conserved in nematodes cultured in solution^22^. On a solid agar plate, *C. elegans* also move in an episodic manner, wherein they crawl actively and persistently in one direction or crawl slowly and stay within a small area, behavioral states called roaming and dwelling/quiescence, respectively^23,25,26^. Individual *Drosophila* flies^8^ and *Leptothorax allardycei* worker ants^27^ also alternate between an actively-moving state and a resting state in an episodic manner. Thus, episodic behavior is a conserved presentation in invertebrates that is thought to be adaptive for supporting food exploration, energy conservation, and reproductive success^8,22,23,28^.

In *C. elegans*, episodic swimming is regulated by *egl-4,* which encodes cGMP-dependent kinase (PKG). PKG is a behavior modulating enzyme conserved across invertebrates and vertebrates^29,30^. *C. elegans egl-4*/*pkg* mutants roam continuously on a solid agar plate and swim when in a solution with less frequent resting than is exhibited by wild-type animals^22,24–26^. Because *egl-4*/*pkg*-dependent regulation is found in both medium conditions^22,24–26^, *C. elegans* episodic motions in both conditions are thought to be regulated by the same molecular/physiological mechanism^23^. In *Drosophila*, the *foraging/pkg* homolog of *egl-4* had been discovered from natural behavioral polymorphisms wherein flies tend to travel long distances (*rover*) or be sedentary (*sitter*)^31,32^. The expression level of *foraging/pkg* differs between *rover* flies and *sitter* flies and *Drosophila* traveling behavior can be switched by genetic manipulation of *foraging/pkg*^33^. Foraging behaviors in social insects—including those exhibited by honey bees (*Apis mellifera*) and ants (*Pheidole pallidula*) that are determined by developmental stage^34^ and social caste^35^, respectively—are also associated with PKG expression and activity. Thus, PKG is considered to be a conserved modulator of animal behavior^29,30^.

To study scaling of behavior across a broad range of time scales, it is necessary to obtain behavioral activity time series of individual animals for an extended period of time at a high temporal resolution. We recorded the swimming behavior of 108 individual *C. elegans* at a semi-video rate for a week-long period by individually culturing the worms in a newly developed microfluidic device. The obtained time series data encompassing 10^7^ time points were submitted to multifractal analysis. *C. elegans* episodic swimming was thus demonstrated to be a scale-free process wherein the transition between actively-moving and inactive states is driven in multifractal kinetics. The specific aim of this study was to employ combinatorial analysis encompassing kinetic modeling and genetic analysis to gain insight into mechanisms that determine motility behavior in *C. elegans*. We conducted kinetic modeling in wild-type *C. elegans* based on multifractal analysis and examination of the sensitivity of the kinetic model in *egl-4/pkg* mutants. We explore the possibility that multifractal *C. elegans* episodic swimming may be derived from nonlinear chemical reactions at a single neuron or a cascadic signaling relay in a multilayered neuromuscular structure. Functional mechanisms by which PKG may modulate multifractal kinetics at the level of a single neuron or in a multilayered neuromuscular structure are also discussed.

## Results

### *C. elegans* episodic swimming is a multi-time scale process

**Figure 1:**
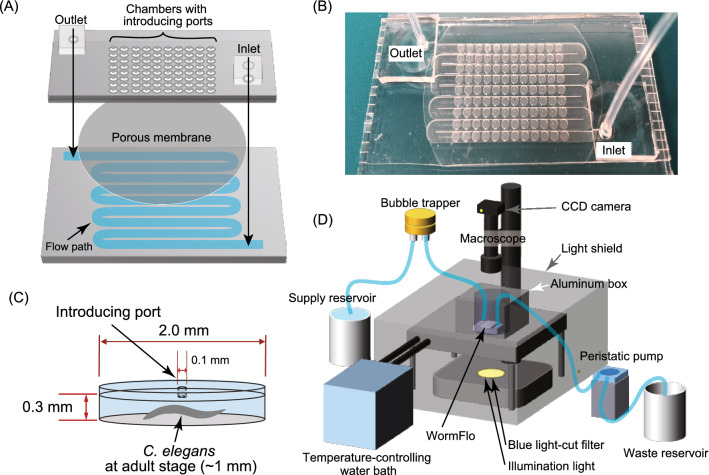
Culturing and recording system for individual *C. elegans* animals. (**A**, **B**) The WormFlo apparatus has a vertically two-compartment structure, wherein an array of 108 culture chambers and loading ports of the upper PDMS chip are partitioned from the buffer flow path located in the lower PDMS chip by a porous membrane (details in the “Methods”). M9 buffer was supplied from the inlet and withdrawn from the outlet. (**C**) The diameter and height of each culture chamber in the upper PDMS chip were 2 mm and 0.3 mm, approximately twofold longer and threefold thicker than the ~ 1-mm-long and ~ 0.1-mm wide *C. elegans* body, respectively. Animals are introduced in each chamber via a 0.1-mm-wide loading port on the roof of a disc-shaped culture chamber, which was closed with a thin PDMS sheet before M9 buffer was perfused. (**D**) *C. elegans* animals cultured in the WormFlo are monitored by a macroscope with a CCD camera. Buffer perfusion is driven by a peristatic pump. To avoid loss of water due to evaporation, the WormFlo was submerged in a 15-cm-diameter glass dish (depth, 5.4 cm) filled with M9 buffer. The temperature of the M9-filled 15-cm glass dish was maintained by circulating temperature-controlled water in a water flow path in the attached aluminum block under the 15-cm glass dish (Fig. S3A). Animals cultured in the microfluidic device are illuminated by blue-light filtered light from a halogen lamp in a light shielding box that buffers against light intensity changes due to daily lab activity.

**Figure 2:**
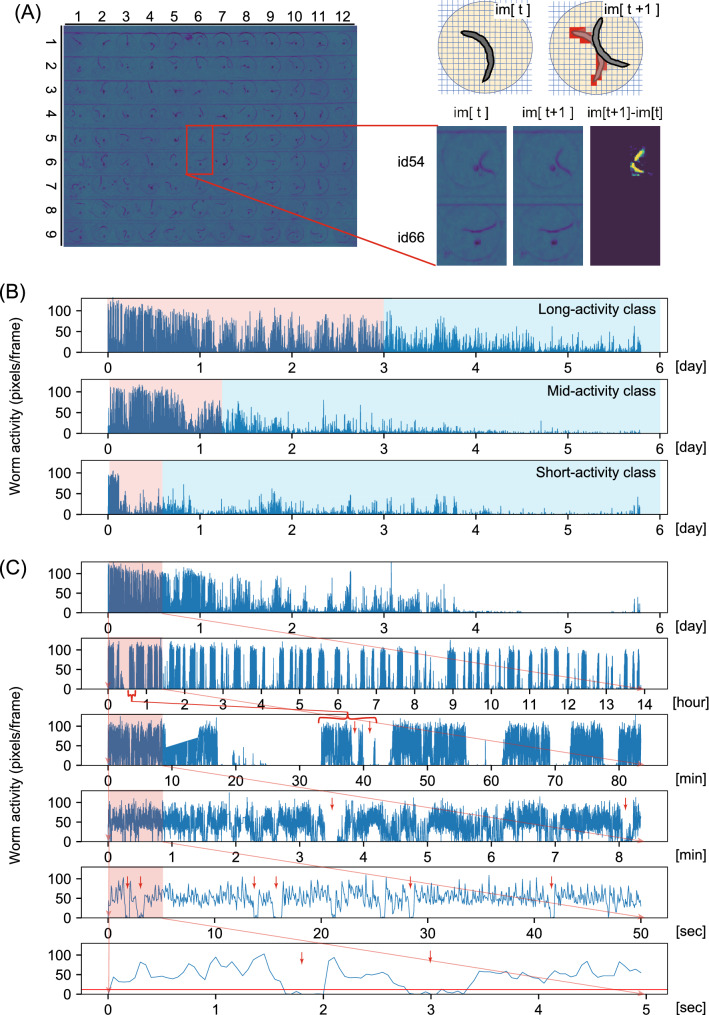
*C. elegans* episodic swimming exhibited a multi-time scale kinetics. Swimming activity of animals cultured in individual chambers was quantified by a pixel counting method (“Methods”). (**A**) Chambers in WormFlo are shown with row-column indexes (left figure). Active pixels (intensity difference > 12 in the range of − 256 to 256) between time point [t] and time point [t + 1] are shown in red pixels in the upper right image and in yellow pixels in the lower right image, respectively. The animal in chamber id 54 moved while the animal in culture chamber id 66 did not move from time [t] to [t + 1]. (**B**) Active pixel numbers are shown on the y-axis as an index of swimming activity with culture time is shown on the x-axis. Six-day temporal activity decay patterns were classified into Long-, Mid-, and Short-activity classes by two criteria; average activities during the early half of the recording period (first 3 days) and the ratio of average activity in the early half to that in the late half of the recording period (see “Methods”). (**C**) Swimming activities of a representative animal in various time scales; full recording time scale (10^7^ timepoints) at the top. Each of the lower panels are a × 10 magnification of the first tenth of its upper panel (red area). Activity threshold at 12 pixels/frame is shown by a red horizontal line in the 5-s scale. Animal activities at 6 day-, 1 day-, 1 h-, 10 min-, 1 min-, and 1 s-scales are shown.

Swimming of multiple adult *C. elegans* individuals was monitored in a newly developed microfluidic device, called WormFlo, which is equipped with an array of 108 disc-shaped chambers for culturing individual *C. elegans* (Fig. 1A,B). *C. elegans* individuals were maintained alone in chambers under controlled chemical, temperature, and light intensity conditions by perfusing M9 buffer without energy source (Fig. 1C,D, Fig. S2, Fig. S3, and “Methods”). We recorded swimming at 10^7^ time points with a 50-ms interval (138 h ≈ 5.8 days), and quantified swimming activity with a pixel counting method (Fig. 2A, Fig. S4, Movie S1, “Methods”, and Supplemental Information). After long term cultivation without energy source, the animals’ swimming activity diminished gradually over time (Fig. 2B and Movie S2). Swimming activities differed markedly between in the early period (high-motility period, red, Fig. 2B) and in the late period (low-motility period, blue, Fig. 2B). The swimming activity in WormFlo chambers decayed with kinetics similar to that seen in animals cultured without energy source in substantially larger (2 × diameter, 23 × volume) 96-well plate wells (Movie S3); and the activity was sustained in animals cultured in WormFlo with energy sources (Fig. S5A and Movie S4). Therefore, the activity decay observed can be attributed primarily to a physiological response to long-term cultivation without energy source rather than to physical damage or spatial restriction.

The length of early high-motility period was highly variable among individuals. Some animals maintained high motility for over half of the entire culturing period (≥ 3 days, Long-activity class, Fig. 2B). Some animals transitioned from high to low motility shortly after the observation (< 1 day, Short-activity class, Fig. 2B). All other animals maintained high motility for an intermediate period (1–3 days, Mid-activity class, Fig. 2B). To analyze time series data, we classified the animals into these three empirical activity classes depending on the length of their early high-motility period (for quantitative criteria, see “Methods”). Consistent with previous studies, we observed episodic swimming bouts on minute to hour time scales (Fig. 2C; 8-min scale and 80-min scale), and a characteristic kinked posture during the resting state in the early high-motility period (data not shown)^22,23^. Interestingly, we observed a swimming bout cluster on a 1 day-scale that could be divided in several clusters in a magnified view of about 1 h (Fig. 2C, compare the red bracketed region in the 14-h scale vs. red arrows in the 80-min scale). This nested temporal structure was observed repeatedly over a series of magnifications from a 1-h scale to a 10-min scale (Fig. 2C, red region in 80-min scale vs. red arrows in 8-min scale), and between the 1-min and 1-s scales (Fig. 2C, red bracket region in 8-min scale vs. red arrows in 50-s and 5-s scales). In the 1-s scale, animals alternated between bending their bodies and beating their bodies for swimming (Movie S1). Series of beating motions were interrupted with intermittent short resting periods, referred as to “posing” (Movie S1). During posing, they were transiently motionless in a bent posture (Movie S1, see legend). The temporal resolution at which continuous shape could be detected in H265 codec-compressed movies was 0.25 s (Fig. S6 and Supplemental Information), which was sufficient to detect subsecond “posing” on a 1-s scale. The temporally nested structure of *C. elegans* episodic motion was observed on time scales of a magnification range of about 1,000 times. Thus, *C. elegans* episodic swimming has multi-timescale dynamics with a self-similar temporal structure.

### A scale-free property in *C. elegans* episodic swimming

**Figure 3:**
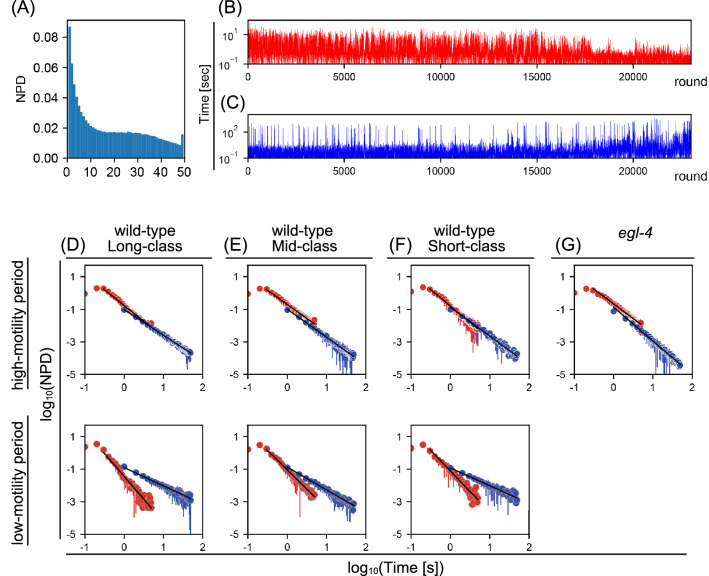
A power law distribution of active/inactive state residence times in *C. elegans* episodic swimming. (**A**) Appearance frequency of number of active pixels in a representative animal for a single image difference frame. Bimodal distribution of active pixels in normalized probability density (NPD) was separated at 10–20 pixels/frame. The active state threshold was ≥ 12 active pixels to obtain residence-time series (details in “Methods”). (**B**, **C**) Residence-time series in the active (**B**) and inactive (**C**) states with the y-axis in log scale. Activity periods above and below the active state threshold (separated by a red horizontal line on 5-s scale in Fig. 2C) were defined as active and inactive periods, respectively. (**D**–**G**) Mean and standard deviations of NPD of residence times in active state (red) and in inactive state (blue) among individual animals in indicated time regime were shown in log–log plot. Fitting was performed over 0.3–5.0 s for active states and over 1–50 s for inactive states. The fit line is shown as a black line. high-motility vs. low-motility periods are as described in “Methods” (wild-type) and the main text (*egl-4* mutants).

Continuous swimming ceased and resumed suddenly (Fig. 2C). Consistently, we observed a bimodal probability distribution of activity strength (Fig. 3A), indicating that *C. elegans* episodic swimming can be characterized by a two-state transition model between actively-moving and inactive states. To reveal the kinetics of the state transition, we studied the statistical distribution of residence times in active and inactive states. Active and inactive states were defined as periods above or below an activity threshold, which was the value in the bimodal distribution valley (Fig. 3A) of swimming activity time series (red horizontal line in Fig. 2C, 5-s scale). Residence time in the two states were obtained alternately from swimming activity time series in a multi-step process. In step 1, the residence time for the first round of active state behavior and subsequent residence time for the first round of inactive state were determined. In step 2, the residence time for the second round of the active state and subsequent residence time for the second round of the inactive state were determined. This process continued such that in step *n*, the residence times for the *n*th round of each of the active and inactive states were determined. Eventually, we obtained *n*-round long residence-time series for each round of active and inactive states (Fig. 3B,C). Residence times ranged from subsecond periods to tens of seconds for the active state and ranged from subsecond periods to hundreds of seconds for the inactive state (Fig. 3B,C).

Residence times in active and inactive states (Fig. 3B,C) were used to obtain probability distributions of residence times. The probability distributions were represented as linear lines in log–log plots or power law distributions (Fig. 3D–F, and Table 1, fit parameter), indicating that the state residence times lack a specific time scale and thus can be described as exhibiting a scale-free property. Comparing the power law exponents between the two states in each activity class, we find that the power law relationship slopes are significantly shallower for the inactive state than for the active state (a “shallow slope” power law relationship indicates a relatively even appearance of longer and shorter residence times, Fig. 3D–F, Table 1 fit parameters and p values in Table 1). These results indicate that the mechanisms that regulate the transition from the active to the inactive state and from the inactive to the active state have distinct scale-free kinetics.

**Table 1:** Summary of fit parameters for power law distributions in Fig. 3D–G and *t* test p-values for the slope of power law distribution fit parameters.

Fit parameters					
		Active		Inactive	
Class	Period	*a*	*b*	*a*	*b*
Long-activity class	High-motility	1.89 ± 0.17	0.81 ± 0.07	1.62 ± 0.13	1.00 ± 0.13
	Low-motility	1.76 ± 0.57	1.52 ± 0.21	1.20 ± 0.23	0.90 ± 0.18
Mid-activity class	High-motility	1.81 ± 0.20	0.77 ± 0.12	1.67 ± 0.20	1.04 ± 0.20
	Low-motility	2.15 ± 0.74	1.30 ± 0.34	1.34 ± 0.24	0.92 ± 0.12
Short-activity class	High-motility	1.87 ± 0.28	0.80 ± 0.17	1.65 ± 0.21	1.06 ± 0.22
	Low-motility	2.06 ± 0.76	1.28 ± 0.34	1.30 ± 0.32	0.93 ± 0.20
*egl-4*(*n479*)	High-motility	2.02 ± 0.43	0.65 ± 0.16	1.96 ± 0.13	1.04 ± 0.19
	Low-motility	–	–	–	–

p-values					
	Active vs inactive		High-motility vs low-motility		
Long activity class	High-motility	7.9 × 10^–6^*	Active	5.70 × 10^–1^	
	Low-motility	2.6 × 10^–2^*	Inactive	5.07 × 10^–4^*	
Mid-activity class	High-motility	3.1 × 10^–3^*	Active	3.03 × 10^–2^*	
	Low-motility	1.2 × 10^–5^*	Inactive	2.61 × 10^–7^*	
Short-activity class	High-motility	4.9 × 10^–6^*	Active	1.50 × 10^–1^	
	Low-motility	8.0 × 10^–7^*	Inactive	1.22 × 10^–7^*	
egl-4(n479)	High-motility	4.2 × 10^–1^	Active	–	
	Low-motility	–	Inactive	–	

			*egl-4*(*n479*) vs Long-activity class		
			Active	1.09 × 10^–1^	
			Inactive	7.79 × 10^–11^*	

(Upper table) Residence time distributions of animals’ active and inactive states in high-motility and low-motility time regimes (defined as in Fig. 2) on a log–log scale fit with y =  − *a*x − *b*. Mean *a* and *b* values are shown with the standard deviations. (Shorter table) *p-*values from t-tests of differences in slope *a* from upper table in indicated comparisons; *p < 0.05.

### Multifractal analysis of numerically-generated data

**Figure 4:**
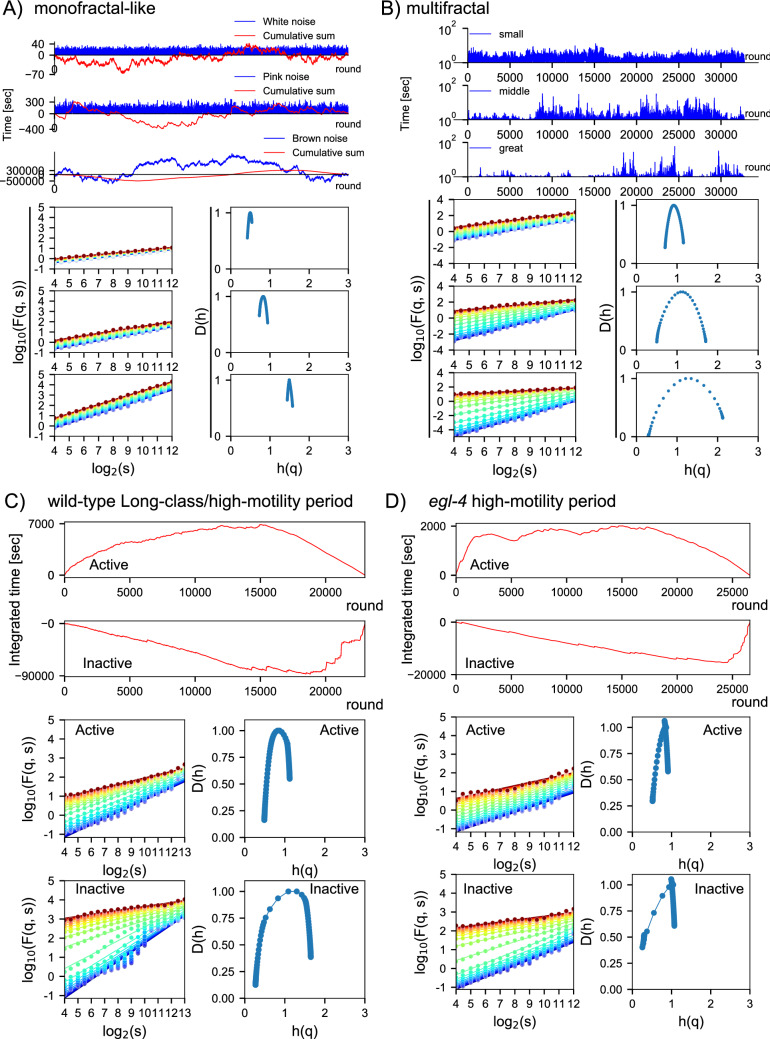
Multifractal analysis of numerically-generated time series and experimentally obtained residence-time series of active and inactive states in a representative *C. elegans* animal. (**A-D**) MF-DFA of numerically-generated time series with monofractal-like (**A**) and multifractal (**B**) properties. The absolute values of white and pink noise time series were shown in (**A**). Time series with small/middle/great multifractality were generated by the multiplicative cascading processes using log-normal functions whose variances were determined by random noises with small/middle/large variances (^41,42^; Supplemental Information) (**B**). Experimentally-obtained active/inactive state residence-time series from high-motility period of Long-activity wild-type (**C**) and *egl-4* mutant (**D**) animals. Time series [blue, in (**A**) and (**B**)] and cumulative sum of the deviations from the average of values in the time series [red, in (**A**), (**C**), and (**D**)] are shown in upper three (**A**, **B**) and two (**C**, **D**) graphs. $F(q,\;s)$ vs. scale *s* plot data (dots) and their fit functions (lines) are shown at lower *q* values (cold colors) and higher *q* values (hotter colors) in a range of − 10 < q < 10 [left column in the lower three (**A**, **B**) and two (**C**, **D**) graphs]; corresponding multifractal spectra are shown in right columns of each graph. Original white, pink, and Brown noise time series are shown, respectively, after 10, 10^2^, and 10^4^-time magnifications to adjust the amplitude of their cumulative sums in (**A**). Note that cumulative sums of noise (**A**, **B**) and residence-time series (**C**, **D**) can take negative values because they are calculated from deviations from the average of the values in the time series. Also note that there is no cumulative sum of the multifractal time series in panel B, because cumulative sums of multifractal time series in log-scale result in a profound loss of information about the smoothness as discussed in cumulative sums shown in a linear sale (**C**, **D**). Instead the intermittent activity bursts in numerically-generated multifractal time series in (**B**) can be compared with experimentally-obtained residence-time series in Fig. 3B,C (both were shown in log scale). The smoothness of the cumulative sums of numerically-generated monofractal-like time series in **A** can be compared with those of experimentally-obtained cumulative sums of residence-time series in (**C**) and (**D**) (both were shown in linear scale).

Residence times were distributed in a scale-free manner (Fig. 3D–F), and long and short bursts of state residence times were clustered rather than uniformly distributed in the residence-time series (Fig. 3B,C); these features are characteristic of multifractal time series. We employed multifractal detrended fluctuation analysis (MF-DFA, “Methods”) to study the temporal structure of the residence-time series data^36–38^. First, time series of white, pink, and Brown noises were numerically-generated by R software tools for fractional Gaussian noise generation^39,40^, and were subjected to MF-DFA. White noise time series is completely uncorrelated, whereas pink and Brown noise time series have long-range correlation. In the time series with long-range correlations, the temporal variation of the cumulatively summed noise time series (i.e. deviation from the trend of cumulative sums) became smoother or more diffusive than that of white noise time series (Fig. 4A; upper three series). The temporal variation, a statistical moment of the cumulatively summed noise time series ($f(v,\;s)$ in Eq. (1)) was estimated by removing local trends of cumulative sums of the time series, by linear fitting to the *v*th local segment of the time series at scale *s* (see “Methods” for detail). To study the scale-dependence of the temporal variation over observation scales, the average of the moments in the cumulative summed noise time series at scale *s* $[\frac{1}{{2N_{s} }}\sum\nolimits_{v = 1}^{{2N_{s} }} {[f(v,\;s)]}$ in Eq. (1), when *q* = 2] was plotted with temporal resolution for observation in *s*. In $F(q,\;s)$ versus *s* plots on a log–log scale, $F(q,\;s)$ increased linearly with increasing scale *s* (Fig. 4A; left column of lower three graphs), as is indicative of fractal scaling^36–38^.

Next, multifractal time series were numerically-generated by multiplicative cascading process^41,42^, and were subjected to MF-DFA. The multifractal time series were highly clusterized, such that the variety of amplitudes and durations of temporal clusters became richer with greater multifractality (Fig. 4B; upper three series). Because of the scale-free and self-similar properties of multifractal time series (Fig. S1 and Supplemental Information), such temporal clusters are distributed in multiple temporal resolutions. To selectively assess fractal property of the temporal clusters with different amplitudes, the temporal variations in the cumulative summed multifractal time series were enhanced or suppressed by the exponentiation factor *q* $[f^{q} (v,\;s)$ in Eq. (1), and “Methods”]. As the variety of amplitudes and durations of temporal clusters increased, the $F(q,\;s)$ versus *s* plot slope in a log–log scale varied widely with the exponentiation factor *q* (compare Fig. 4A,B; left column of lower three graphs). The *q*-dependent change in slope in a log–log scale $F(q,\;s)$ versus *s* plot is indicative of multifractal scaling.

To evaluate *q*-dependent change of fractal property in multifractal time series, the slopes of $F(q,\;s)$ versus *s* plot gave rise to a multifractal spectrum, showing a relationship between *q*-order local Hurst exponent (Hölder exponent *h*(*q*)) vs. *q*-order singularity dimension (*D*(*h*))^36–38^ (see “Methods”). The *q*-order singularity dimension is a fractal dimension that represents time series sparsity of *q*-order temporal variations; a *D*(*h*) < 1 represents sparsely distributed local structures of the entire time series, and a *D*(*h*) approaching 1 representing broadening involvement of the entire time series (Supplemental Information). Thereby, local Hurst exponent at the peak of multifractal spectrum at around *D*(*h*) = 1 represents Hurst exponent of a global structure of the time series (global Hurst exponent, *h*_*peak*_), whereas a width of multifractal spectrum represents a rich variety of local structures that exhibit various local Hurst exponents. Thus, multifractal spectrum is characterized by the peak and the width of the spectrum.

Mono-fractal and multifractal time series have extremely slowly decaying autocorrelations called long-range memory^43,44^. Long-range memory makes diffusive trajectory with apparent upward or downward trends in cumulative sums of fractal time series due to a high tendency where a positive (negative) increment follows positive (negative) increments over long-range. Because white, pink, and Brown noise time series have no, weak, and strong tendency/memory of the increments, cumulative sums of white, pink, and Brown noise time series accordingly have more diffusive trajectory and *h*_*peak*_ values of them become larger. Therefore, the global Hurst exponent *h*_*peak*_ is an index of time series memory. In numerically-generated monofractal-like time series, as the time series memory became stronger, the slope of $F(q,\;s)$ versus *s* plot on a log–log scale increased (Fig. 4A; left column of lower three graphs), and accordingly *h*_*peak*_ values for the cumulative sum of these noise time series became larger (Fig. 4A; right column of lower three graphs). The *h*_*peak*_ values were estimated to be approximately 0.5, 1, and 1.5 (Fig. 4A; right column of lower three graphs), which were consistent with theoretical Hurst exponents of cumulative sums of these noise time series^36,37^. On the other hand, a wide multifractal spectrum indicates that the multifractal time series contain a rich variety of locally clustered structures with various local Hurst exponents. Therefore, multifractal spectrum *width* in animal activity time series is an index of behavioral complexity. In numerically-generated multifractal time series, as multifractality becomes greater, the *q*-dependent variation of the slopes became wider (Fig. 4B; left column of lower three graphs), and the width of multifractal spectrum (*width*) became larger significantly without a substantial change in *h*_*peak*_ (Fig. 4B; right column of lower three graphs). Thus, global Hurst exponent *h*_*peak*_ and multifractal spectrum *width* are independently changeable in a scale-free time series. For more information of numerical generation for noise time series and multifractal analysis, and for an introduction into fractal scaling, see the Supplemental Information.

### Episodic swimming is driven by two-state transition with two distinct multifractal kinetics

**Figure 5:**
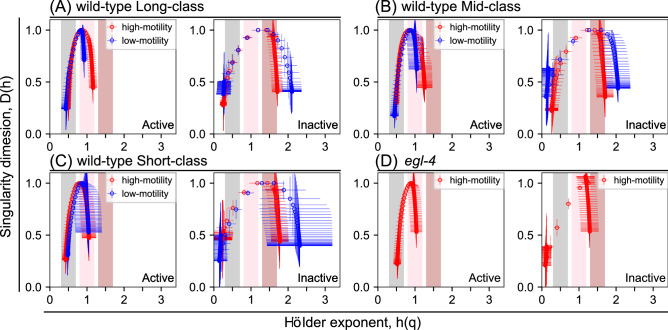
Multifractal spectra averaged from multiple animals. Multifractal spectra mean (and standard deviations) were calculated from residence-time series data spectra of active (left) or inactive (right) states from multiple wild-type animals in Long- (**A**), Mid- (**B**), and Short- (**C**) activity classes in the high-motility (red circles) or low-motility (blue circles) periods. For *egl-4* mutants (**D**), spectra represent only in high-motility (red circles) time regime. Note that the spectrum *width* in high-motility period was as narrow as in low-motility period in Short-activity classes, which was due to too short high activity period during the defined high-motility period among animals in Short-activity classes (**C**). Grey, pink, and brown areas in the graphs indicate the x-axis positions for the global Hurst values of white, pink, and Brown noise, respectively.

Global trend in the residence-time series data of active- and inactive-states became gradually shorter and longer, respectively, after transition from the early high-motility to the late low-motility periods (~ 15,000 rounds; Fig. 3B,C). The temporal variations of cumulative sums, or the deviations from the trend appeared to differ qualitatively between active and inactive states; that of active states was smoothly curved, whereas that of inactive states was locally straighter in the high-motility period (Fig. 4C), suggesting that noise properties of active and inactive states qualitatively differed. In the log–log scale $F(q,\;s)$ versus *s* plot, $F\left( {q,s} \right)$ in active and inactive states increased almost linearly with *s* and the slope varied with *q* (Fig. 4C; left column of lower two graphs), and the multifractal spectra of active state and inactive states in the high-motility and low-motility periods were widely distributed (Fig. 4C; right column of lower two graphs and Fig. 5), indicating that the residence-time series of active and inactive states had a multifractal nature.

**Figure 6:**
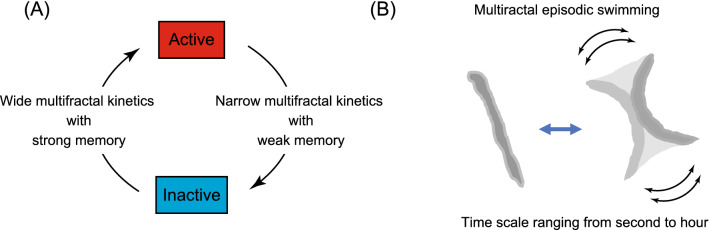
Multifractal episodic swimming in *C. elegans.* (**A**) Alternate state transitions are driven by distinct kinetics: active-to-inactive and inactive-to-active state transitions follow distinct multifractal kinetics. (**B**) Because *C. elegans* episodic swimming is driven by alternating transitions between an actively moving state and an inactive (resting and posing) state over a broad range of temporal scales (i.e. seconds to hours) and the residence-time series data of which are characterized by a multifractal nature, we refer to this behavior as “multifractal episodic swimming”.

The global Hurst exponents *h*_*peak*_ in multifractal spectra of active and inactive states were located around *h*(*q*) = 1, indicating that the state residence times have a long-range temporal correlation, as is seen in pink noise time series. As shown in Fig. 5A–C, in both of high-motility and low-motility periods in all activity classes, we observed greater *h*_*peak*_ values for the inactive state (average, 1.38) than for the active state (average, 0.85) as well as greater *width* values in the inactive state (average, 1.80) than in the active state (average, 0.64), all p < 0.05). Hence, residence-time series revealed distinct multifractal properties between active and inactive states, with a longer behavioral memory and greater behavioral complexity for the inactive state than for the active state. We called kinetics that generate scale-free residence times with temporal correlation and temporal clusterization as multifractal kinetics. Accordingly, we were able to explain *C. elegans* episodic swimming with a two-state transition model in which opposite transitions between actively-moving and inactive states are driven by distinct multifractal kinetics (Fig. 6A).

The power law exponent showed a more pronounced alteration between in high-motility and low-motility periods in residence-time series for the inactive state (average, 22.4%) than for the active state (average, 7.0%). The change in the power law exponent was only statistically significant for the inactive state (p values and fit parameters in Table 1). MF-DFA showed that the global Hurst exponent *h*_*peak*_ was not altered significantly by the transition to the low-motility period in either the active state (average high-motility *h*_*peak*_, 0.86; average low-motility *h*_*peak*_, 0.84) or the inactive state (average high-motility *h*_*peak*_, 1.34; average low-motility *h*_*peak*_, 1.42) (*h*_*peak*_ values, and p values in Table 2, all p > 0.05). However, the multifractal spectrum *width* was altered in the low-motility period, becoming narrower (− 17.1%) in the active state and wider (+ 31.6%) in the inactive state (*width* values, and p values in Table 2, all p < 0.05 except active state in Short-class, see Fig. 5 legend). These results indicate that food signaling or metabolic state regulates behavior by modulating multifractal kinetics after long-term cultivation without energy source. That is, in the late low-motility period associating with the depletion of energy stored in the body, the animals do not simply decrease swimming activity, but show a selective modulation of multifractal kinetics.

**Table 2:** Summary of *h*_*peak*_ and *width* values of the multifractal spectra in Fig. 5A–D and associated t-test p-values.

*h*_*peak*_ and *width*						
Class	Period	Active		Period	Inactive	
		*h*_*peak*_	*Width*		*h*_*peak*_	*Width*
Long-activity class	High-motility	0.89 ± 0.05	0.67 ± 0.11	High-motility	1.39 ± 0.14	1.49 ± 0.19
	Low-motility	0.83 ± 0.09	0.50 ± 0.14	Low-motility	1.34 ± 0.39	1.88 ± 0.29
Mid-activity class	High-motility	0.88 ± 0.09	0.77 ± 0.21	High-motility	1.30 ± 0.31	1.51 ± 0.20
	Low-motility	0.88 ± 0.13	0.62 ± 0.19	Low-motility	1.34 ± 0.60	2.00 ± 0.35
Short-activity class	High-motility	0.80 ± 0.10	0.65 ± 0.18	High-motility	1.33 ± 0.36	1.65 ± 0.41
	Low-motility	0.81 ± 0.18	0.61 ± 0.32	Low-motility	1.59 ± 0.33	2.25 ± 0.81
*egl-4*(*n479*)	High-motility	0.88 ± 0.07	0.53 ± 0.20	High-motility	1.13 ± 0.20	1.28 ± 0.25
	Low-motility	–	–	Low-motility	–	–

	p-values					
		Active			High-motility vs low-motility	
		*h*_*peak*_	*Width*		*h*_*peak*_	*Width*
Long-activity class	High-motility	9.52 × 10^–13^*	7.51 × 10^–16^*	Active	1.36 × 10^–1^	1.06 × 10^–2^*
	Low-motility	6.50 × 10^–3^*	8.16 × 10^–8^*	Inactive	7.07 × 10^–1^	5.33 × 10^–3^*
Mid-activity class	High-motility	1.00 × 10^–6^*	2.38 × 10^–16^*	Active	8.33 × 10^–1^	2.33 × 10^–2^*
	Low-motility	6.70 × 10^–3^*	3.61 × 10^–13^*	Inactive	8.06 × 10^–1^	3.70 × 10^–5^*
Short-activity class	High-motility	1.00 × 10^–5^*	3.18 × 10^–9^*	Active	8.74 × 10^–1^	7.76 × 10^–1^
	Low-motility	2.00 × 10^–6^*	1.91 × 10^–5^*	Inactive	5.92 × 10^–2^	3.27 × 10^–2^*
egl-4(n479)	High-motility	4.45 × 10^–9^*	1.98 × 10^–23^*	Active	–	–
	Low-motility	–	–	Inactive	–	–

				*egl-4*(*n479*) vs wild-type Long		
				*h*_*peak*_	*Width*	
			Active	5.42 × 10^–1^	1.15 × 10^–3^*	
			Inactive	1.00 × 10^–6^*	1.16 × 10^–3^*	

(Upper table) Hurst exponent for the entire time series (*h*_*peak*_) and widths of multifractal spectrum (distance of local Hurst exponent at − 10 < *q* < 10) obtained from individual animals in the indicated activity class or mutant animals in high-motility and low-motility time regimes. (Shorter table) p*-*values from *t* tests of differences in *h*_*peak*_ and *width* from upper table in indicated comparisons; *p < 0.05.

### PKG may regulate the multifractal kinetics of *C. elegans* episodic swimming

For molecular dissection of the multifractal kinetics *C. elegans* episodic swimming, we studied the *egl-4*(*n479*) mutant, in which the temperature sensitive *n479* allele of *egl-4* is associated with a defect in conventional episodic swimming characterized by lengthened continuous swimming periods and a reduced frequency of resting^22,24^. We confirmed that *egl-4*(*n479*) mutants had reduced frequencies of a persistent inactive state in our quantitative activity time series (Fig. S5B). The *egl-4*(*n479*) mutants cultured in WormFlo without energy source were too transparent for body detection in the later portion of the 6-day culture period. This transparency was likely caused by the mutants’ unusually early lipid consumption due to their continuous swimming and lack of long resting; lipids in worm bodies scatter illuminating light enabling imaging as a (relatively) dark object as shown in Fig. 2A. Thus high-contrast images of the first tenth of the 6-day culturing period were used for analysis of the high-motility period of *egl-4* mutants.

In *egl-4* mutants, the power law relationships were maintained in active and inactive states, but the power law exponent was preferentially changed in the inactive state (+ 21.0%) compared to that in active state (+ 6.9%) (Fig. 3D,G). Power law exponents differed significantly between wild-type worms and *egl-4* mutants only in the inactive state (p-values and fit parameters in Table 1), indicating that the mutant has altered residence times across time scales rather than a defect that is specific to a particular time scale. MF-DFA showed that the shape of the multifractal spectrum in *egl-4* mutants was largely altered in the inactive state (Figs. 4D, 5D). Comparing the global Hurst exponent between *egl-4* mutants and Long-activity class wild-type worms, we found that active-state *h*_*peak*_ was similar between the groups (difference, 1.1%), whereas inactive-state *h*_*peak*_ differed significantly between the groups (difference, 18.7%). Meanwhile, spectrum *width* in *egl-4* mutants was reduced significantly relative to that obtained for the Long-activity wild-type group in both active (− 20.9%) and inactive (− 14.1%) states (*h*_*peak*_ and *width* values, p values in Table 2). Thus, *egl-4* mutants showed a defect in both temporal memory and temporal clusterization in the inactive state, but showed a defect in only temporal clusterization in the active state. These results are consistent with the possibility that EGL-4/PKG may regulate the multifractal kinetics of behavior, and, more specifically, suggest that EGL-4/PKG may regulate the active- and inactive-state kinetics of *C. elegans* episodic swimming differently.

## Discussion

### *C. elegans* episodic swimming is driven by a multifractal transition cycle

Experimental measurements of temporal changes in biological system variables of interest and the statistical analysis of those data can provide information about the hidden operating principles of that system^45^. *C. elegans* episodic swimming was characterized conventionally by the average residence time of active- and inactive-states^22–24,46^. Here, we applied multifractal analysis to study the molecular and genetic mechanisms regulating animal behavior about a 6-day period recorded at a subsecond temporal resolution, showing that episodic swimming is a scale-free process (Fig. 2C). Power-law/scale-free distributions, such as those shown in Fig. 3D–G, have a right skewed and extremely long-tailed distribution. The averages of fit functions of such distributions can be enormous in value or approach infinity, whereas the averages determined experimentally will vary greatly depending on the presence or absence of low-frequency huge values in the experimentally obtained dataset. Thus, this “average” is not a robust experimental parameter that can be used to characterize scale-free distributions^47^. Instead, the scale-free process was characterized by a power law exponent in the relationship between appearance frequency and residence time (Fig. S1A and Supplemental Information). Scale-free residence-time series that have a long-range memory and local complexity are characterized by a multifractal process. Hence, we referred to the swimming as “multifractal episodic swimming” (Fig. 6B).

A scale-free distribution of actively-moving and inactive residence times has been reported for episodic *Drosophila* behavior; modeling suggested that the scale-free nature of these residence times contributes to maximizing the food exploitation area while optimizing food intake time^8^. Previous analyses have shown long-range temporal correlations in scale-free properties of the behaviors of many species, including fractal (but not multifractal) analysis of *C. elegans* crawling^6,7,9–12^. The fractal nature of *C. elegans* crawling in agar^9^ is consistent with our long-range memory and clusterization finding. In this study, we extended previous findings by combining a two-state transition model with multifractal analysis. Our analysis indicated that *C. elegans* episodic swimming is characterized by a two-state transition between actively-moving and inactive states, wherein the two transitions are driven by distinct multifractal kinetics. That is, the active-to-inactive transition is driven by a narrow multifractal kinetics with weak long-range memory, whereas the inactive-to-active transition is driven by a wide multifractal kinetics with strong long-range memory (Fig. 6A).

Multifractal episodic motion of *C. elegans* in solid and liquid environments may be an adaptation to food environments. The colonies of bacteria that *C. elegans* worms feed on grow in a fractal shape^48^. In *C. elegans*, the actively-moving state is likely to enable food foraging, whereas the inactive state is likely associated with food intake, egg-laying, or resting to save energy or satiety^23,28–30,49,50^. Temporal correlation in the inactive state gives rise to a series of long and short periods for food intake that may follow the fractally-shaped bacterial colony^48^, whereas temporal correlation in active state residence-time series gives rise to a series of long- and short-distance foraging bouts that may follow the interbranch distances of a fractally-shaped bacterial colony. Thus, the scale-free and temporally structured residence times for food foraging and intake may be adaptive to the fractal shape of bacterial colonies. *C. elegans* survival strategies are altered by food availability. After a long-term cultivation without energy source, *C. elegans* saves their energy for long-distance foraging and instead spend more effort for balancing resting and food intake at a local area, e.g. through reuptake of their excrements. Food-dependent modulation of multifractal kinetics of behaviors may improve food intake efficiency and reproductive success in natural environments. This possibility should be tested in a modeling study.

Our quantitative studies showed that *egl-4* mutants exhibited different alterations of multifractal kinetics in the active versus the inactive state. This result suggests that EGL-4 may regulate the multifractal kinetics of animal behaviors. However, *egl-4* mutants’ defects in behavioral memory and behavioral complexity were smaller in magnitude than the differences between the active and inactive states in wild-type *C. elegans*, and also smaller than the index that characterizes qualitative differences of noise properties among white, pink, and Brown noises. Differences between active and inactive states in wild-type *C. elegans* were comparable to or greater than the index (compare *h*_*peak*_ in Table 2: *h*_*peak*_ of white, pink, and Brown noises differ by 0.5). Therefore, multifractal kinetics in *egl-4* mutants were within the range of kinetics qualitatively same as those in wild-type. In addition, these defects may be caused by a behavioral variation of the *egl-4* mutant strain rather than by loss of function of *egl-4/pkg* per se. It is necessary to apply other fractal analyses and further molecular/genetic studies to examine these possibilities.

### PKG-modulated multifractal transition may be widely applicable to multi-time scale behaviors

Multifractal transition cycle and its PKG-dependent modulation may be shared among many invertebrates. In short-term (sub-minute) observations, *Drosophila*^8^ and *Leptothorax allardycei* worker ants^27^ exhibit bimodal behavioral mode switching^8,27^ similar to that in *C. elegans*. Notably, *Drosophila* residence times in actively-moving and inactive states in a long-term observation period were distributed in a scale-free manner^8^. Interestingly, a bimodal behavioral choice between long-distance moving foragers and dwellers has been shown to be regulated by PKG in *C. elegans*^25,26,51^, *Drosophila*^31^, bees^34^, and ants^35,52^. Both *C. elegans* mutant^25,26^ and *Drosophila* polymorphism^31,53^ local dwellers spend more time on local food intake/resting. Local dwellers conduct brood care in bee hives^34^ and ant nest defense^35,52^. Forager and dweller behavioral phenotypes are switched developmentally in bees, but maintained through the lifespan in the ant caste system. In *C. elegans*^25,26,51^ and ants^35^, long-distance foraging is associated with low PKG activity or expression, whereas local dwelling is associated with high PKG expression. Conversely, long-distance foraging (local dwelling) is associated with high (low) activity or expression of PKG in flies^31^ and bees^34^. The time scale difference of PKG-active periods between ants and honey bees, and the reversed function of PKG for long-distance foraging or local dwelling between nematodes/ants and flies/bees may reflect a heterochronic evolutionary change in the developmental control of PKG expression^34^, and an evolutionary adaptation of the PKG signaling system in molecular drivers of behavior, respectively. Despite evolutionary changes in time scale and the functional role of PKG, these results indicate that appearance frequency for actively-moving and inactive states is modulated by PKG. Although the role of PKG in the episodic motions of *Drosophila* and ants has not been studied directly, the aforementioned findings suggest that at least episodic motions in *Drosophila* and ants may be regulated by multifractal kinetics and its PKG-dependent modulation.

Surprisingly, PKG-dependent modulation of the transition kinetics in a multifractal transition cycle may be involved in human heartbeat physiology. Electrocardiogram time series^54–56^ of cardiac muscle depolarization and repolarization are characterized by multifractality. The inter-beat (RR) interval is the period between peaks of R waves, which reflect ventricular depolarization. The intra-beat (QT) interval is the period from the peak of Q wave to the end of T wave, which corresponds to the depolarization in the left side of the intraventricular septum and the repolarization of ventricular muscles, respectively. RR- and QT-interval series are characterized by a multifractal structure^57^. Interestingly, the shape of the multifractal spectrum for RR intervals differs from that for QT intervals, raising the possibility that QT and RR intervals may be regulated by distinct multifractal kinetics. PKG has been shown to regulate the heartbeat in mice and flies^58–61^. PKG-knockout mice^59^ or cardio-myocyte specific PKG-knockout mice^61^ show hypertension. Although the mechanism is still in dispute^60^, the defects in these knockout mice indicate that PKG is involved in the relaxation phase of the cardiac cycle. Thus, the heartbeat can be regulated by a multifractal transition cycle and is subject to PKG-dependent modulation, similar to that we documented for *C. elegans* episodic swimming. Thus, PKG-modulated multifractal transition cycles may occur in various animal species, including humans, and organs.

### Molecular and system-level mechanisms for multifractal episodic swimming

Based on previously reported models designed to recapitulate scale-free and multifractal time series, we developed hypotheses for molecular and system-level mechanisms underlying the multifractal nature of animal behaviors, and discuss how PKG may be involved in the mechanism. First, the intermittent bursts of a single neuron can be reproduced in chaos dynamical systems^62–68^; owing to their non-Gaussian fluctuation with intermittency, such systems may be associated with multifractality in animal behaviors. Chaotic dynamics reproduced in these models are generated by interactions between fast inflows and slow outflows of ions to/from a neuron. Thus, multifractal nature in behavior might be attributable to chaotic biochemical reactions in a single neuron. Second, when a system is in a certain state, called the criticality, local interactions among system components related to order and noise for randomization of the order reach a critical balance and, eventually, self-similar dynamics emerge spontaneously in the system^69,70^; scale-free dynamics due to the criticality can be associated with multifractality^71,72^. Experimentally observed scale-free neural activity in intact brains was recapitulated in self-organized criticality-based models that assumed global interaction of neuronal signals in a hierarchically structured neural network representative of brain structures in *C. elegans* and humans^73,74^. Therefore, multifractality in animal behaviors may be attributed to criticality in the hierarchal structure of brains. Third, although implications of molecular and physiological mechanisms in the multifractal nature of animal behaviors have not been reported, the multiplicative cascade model is a simple mathematical model that generates multifractal time series (Fig. 4B and Supplemental Information)^41,42,75,76^. Generally, multiplicative cascade models generate multifractal time series via a series of multiplications of log-normal random noise in a cascade manner, enhancing time series variance greatly as it progresses (Supplemental Information). It has been shown that waiting time distributions of neuronal action potentials exhibit a log-normal distribution consequent to the nonlinearity of reaction systems in neurons^77–79^, whereas hierarchal multiplication may correspond to a cascading signal relay in the brain. Thus, multifractal nature in behaviors may be attributed to both non-linear reactions within neurons and global interaction in hierarchal structures of neural networks. To reveal molecular- and system-level mechanisms of *C. elegans* multifractal episodic swimming, it will be critical to identify the operating principles that are functioning in multifractal episodic swimming in *C. elegans*. With regard to PKG, selective ectopic expression of PKG in R3 and R4d ring neurons is sufficient to restore behavioral defects in *Drosophila* with *pkg*/*foraging* alleles^80,81^. Additionally, differential PKG expression was identified in a set of five specific neurons between different castes of ants^35^. In *C. elegans*, PKG functions in a limited number of neurons^24^. PKG has been shown to regulate synaptic vesicle cycling, Ca^2+^ influx via G-protein signaling, and axon guidance in certain neuron types^80,82–85^. The endogenous and ectopic expression of PKG in specific neurons and functional analysis of PKG suggest that PKG modulates multifractal kinetics of animal behaviors in single neurons or small numbers of neurons. Thus, multifractal kinetics generated in local pacemaker neurons or global hierarchal structures of neural networks may be modulated by a local interaction with PKG-regulated neurons.

In this study, our experiments revealed a basic kinetic mechanism of *C. elegans* multifractal episodic swimming. The findings are applicable to diverse fields of interest, including human heartbeat physiology. Due to the wide variety of molecular and genetic tools available for *C. elegans* research, our observation and analysis approach may be used to help reveal conserved mechanisms underlying the multifractal nature of animal physiology and behavior.

## Methods

### *C. elegans* strains used and their maintenance

The Bristol N2 strain was used as wild-type *C. elegans*. N2 and *egl-4*(*n479*) mutant animals were maintained on agar plates with *E. coli* OP50 strain at 15 °C^86^.

### Design, fabrication, and characterization of microfluidic device

To maximize the number of animals monitored in the recording area, our device employs a two-vertical-compartment structure with the array of culture chambers located over a flow path, whose boundary was partitioned by a porous membrane (Whatman 111115, Nuclepore Hydrophilic Membrane, 10-µm pores, GE Healthcare, USA) (Fig. 1A,B). The upper polydimethylsiloxane (PDMS) chip consisted of a 108-chamber array (1 worm/chamber) with buffer-inlet and exchange solution-outlet ports in the chambers. The lower PDMS chip consisted of a snake-shaped microchannel for supplying liquid buffer to the culture chambers. PDMS chips were fabricated by conventional replica molding with the SU-8 epoxy-based photoresist^87,88^. Animal-loading ports (100 µm wide) on the top of each of chamber in the upper PDMS chip were made with a Zing 16 laser (Epilog Laser, Japan). Before culturing and buffer exchanges, the loading ports were sealed with a PDMS sheet. Each culture chamber (2-mm diameter and 0.3-mm height) in the upper PDMS chip was approximately twofold-longer and threefold-thicker than the ~ 1-mm-long and ~ 0.1-mm-wide *C. elegans* body (Fig. 1C). The serpentine buffer supply microchannel in the lower PDMS chip had a 2.2-mm diameter, covering each upper-chip culture chamber. The three parts (upper and lower PDMS chips, and the microporous membrane) were assembled by covalent bonding with an aminosilane coupling agent and oxygen plasma treatment^89^.

### Culturing *C. elegans* in a microfluidic device

Animals at the young adult stage were collected from an agar plate. After an agar plate-culture period (2 days at 24 °C for wild-type and 3 days at 15 °C for *egl-4* mutants), the animals were collected manually in room-temperature M9 buffer and introduced into the microfluidic device by manual pipetting via chamber loading ports (Fig. 1C). With previous devices, individual *C. elegans* were held in a clump structure and released into the chamber by applying deforming high pressure^90–92^. We instead used manual pipetting to avoid applying mechanical stress during loading. To maintain a constant chemical environment in the culture chambers, M9 buffer (with or without 1 g/L glucose and 5 mg/L cholesterol) was perfused continuously with a peristatic pump (Fig. 1B,D) at a flow rate at 5 mL/h. Flow rate was measured by monitoring weight changes in the water discharged from the outlet (data not shown). Buffer exchange in chambers with the flow passing through the porous membrane was confirmed in experiments with a fluorescent solution (Fig. S2). Air bubbles in the buffer supply tube were removed with polytetrafluoroethylene membrane in an Omnifit bubble trap (006BT, Diba Industries Inc., USA) (Fig. 1D).

### Observation of *C. elegans* swimming

*C. elegans* behaviors were recorded at 20 frames/s through a macroscope with an apochromat objective lens (1 ×) (Z16 APO, Leica, Germany) and a CCD camera (1,940 × 1,460 pixels, 2.8 Megapixel) with a USB3.0 connection (MD028MU-SY, Ximea, Germany). The camera was controlled by Micromanager (https://micro-manager.org). Movies were compressed (H265 codec in FFmpeg) every 10,000 frames (500 s) into mp4 files. *C. elegans* are sensitive to temperature change of 4 °C^93^ and highly sensitive to blue-ultraviolet light, but not to green/yellow light (> 545 nm)^94, 95^. Temperature of culture chambers on WormFlo were maintained by submerging WormFlo in M9 buffer in a 15-cm-diameter glass dish, whose temperature was maintained by temperature-controlled water supplied from a high-precision water bath (HAAKE, Germany) (Fig. 1D and Fig. S3A and B). A temperature logger (TC-08, Pico Technology, UK) confirmed that the temperature of the M9 buffer in the glass dish was maintained within ± 0.5 °C during the 6-day recording period (Fig. S3C). The recording system was covered by a light shield to prevent illumination changes from light fluctuations related to daily lab activities (Fig. 1D). Blue light of illumination light from a halogen lamp was filtered out with a 0.5-mm-thick orange acryl plate (Fig. 1D and Fig. S3A); spectrophotometry (USB400, Ocean Optics, USA) confirmed that wavelengths < 500 nm were filtered out (Fig. S3D). Standard deviation of light intensity change at the culture chambers during observation period was kept within 10% of the average (Fig. S3E). This culturing and recording system allowed us to monitor individually cultured *C. elegans* in an environment with minimized chemical, light, and temperature perturbations.

### Quantification of *C. elegans* swimming activity

We measured *C. elegans* swimming activity by counting the number of pixels with an intensity over a certain threshold in a matrix obtained from the difference between intensities obtained at t and t + 1 (we referred to it as image difference) using the Open CV module in Python2.7 (Pixel counting method). By employing a pixel counting method, information contained in the original movies (e.g. worm postures and postural dynamics) were reduced into one-dimensional time series that provide an index of behavioral activity (Fig. 2). When an animal moves at a frame interval, the dark pixels detecting its body in image[t] become brighter in image[t + 1], yielding an increased image difference (= image[t + 1] – image[t]) (Fig. 2A). For pixel counting, the image difference matrix values were in the range of – 256 to + 256 at 1,940 × 1,460 pixels. The pixels with intensity differences greater than 12 value within this range thus exhibit an image difference and are counted as “active” pixels [note that the pixel intensity threshold of 12 is different from the activity threshold (12 pixels/frame) to define residence time in active and inactive states (Fig. 3A)]. The pixel intensity threshold of 12 was determined empirically so that actual movements of animals are detected efficiently while avoiding artefact-based false hits due to thermal noise in a pixel on the camera sensor. Based on pixel counts, the size of the area the animal moved through during each time frame (50 ms) was measured (Fig. 2A). Image and data processing, including compensation for artifactual activity in the time series are described in the Supplemental Information (Fig. S4 and S6). We classified animals into three classes; for the Long-activity class, the high-motility and low-motility periods were defined by early 50% period (≥ 3 days) from the start of recording through the early half of the recording period and the remainder of the period: for the Mid-activity class, high-motility period and low-motility period were defined by the period from the start of recording to early 20% period of the recording period (1–3 days) and the remainder: for the Short-activity class, the high-motility and low-motility periods were defined by the period from the start of recording to early 10% of the recording period (< 1 day) and the remainder. To classify animals, we measured average activities during the early half of the recording period (day 0 to day 3; abbreviated “AA0-3”) and the ratio of AA0-3 to average activities during the latter half of the recording period (day 3 to day 6; abbreviated “AA3-6”). Animals with high AA0-3 and a low AA0-3 to AA3-6 ratio were classified as Long-activity. Animals with high AA0-3 and a high AA0-3 to AA3-6 ratio were classified as Mid-activity. Animals with low AA0-3 and a low AA0-3 to AA3-6 ratio were classified as Short-activity. The threshold of high AA0-3 was set empirically at 0.75 and the threshold of a high ratio was set to 0.01. Note that 0.75 threshold values are low due to the bursty/sparse nature of *C. elegans* swimming activity.

### Data analysis

Active-state residence time was defined as the period after the start of the activity burst to the end of the burst; inactive-state residence time was defined as the period starting immediately after the end of activity burst to the next round of activity burst in the activity time series. Alternating-state residence-time series were obtained by thresholding time series of swimming activity at 12 pixels/frame, which corresponded to the valley of a bimodal activity distribution (Fig. 3A).

MF-DFA was performed in Python software^96^ using

1 $$F(q,\;s) = \frac{1}{{2N_{s} }}\left\{ {\mathop \sum \limits_{v = 1}^{{2N_{s} }} \left[ {f^{2} (v,\;s)} \right]^{q/2} } \right\}^{1/q}$$

where $f(v,\;s)$ is the temporal variation or fluctuations from the local trend of cumulative sums of the deviation of residence time from the average residence times in the *v*th segment at the temporal resolution for observation $(s)$. MF-DFA was derived from detrended fluctuation analysis (DFA)^97^; the equation used in DFA corresponds to the equation used in MF-DFA when $q = 2$ in Eq. (1). In operation 1, the entire cumulative sum series was segmented into *Ns* segments, and the local trend in each *v*th segment was determined by piecewise fitting with a linear function. When the entire time series are segmented over a certain length (equivalent to scale *s*) (from the beginning to a defined endpoint), the last one segment is left as a flanking fragment at the end of the time series. To include the end-flanking fragment in the analysis, segmentation was started in turn from the end of the time series. In total, 2 × $N_{s}$ segments were obtained to calculate $F(q,\;s)$. In operation 2, the amplitude of fluctuations from the local linear trend at each segment was enhanced (or suppressed) to a large (or small) amplitude of $f(v,\;s)$ by exponentiating with positive (or negative) *q*-values; *q* value-exponentiated $f(v,\;s)$ was summed over all the *v*th segments. $F(q,\;s)$ versus *s* plots were log–log plotted, and the slope at each *q*-value was fit with a linear function. Finally, multifractal spectrum [*q*-order (local) Hurst exponent (Hölder exponent), *h*(*q*) vs. *q*-order singularity dimension, *D*(*h*)] was obtained from $F(q,\;s)$ vs. *s* plots at each *q*-value by using $h(q) = H(q) + q\frac{dH(q)}{{dq}}$ and $D(h) = q(h(q) - H(q)) + 1$ ($H(q)$ is generalized Hurst exponent, corresponding to the slope of $F(q,\;s)$ vs. *s* plots at each *q*-value)^36–38^. Linear fitting to data in a log–log plot minimizes relative error between the fit function (y_fit) and the data (y_data), which is log(y_fit/y_data) (= log(y_fit)-log(y_data)). Linear fitting to data in a log–log plot avoids biased fitting due to errors being weighted by high-value data points that happens when fitting is performed by minimizing absolute error (y_fit – y_data). Animals whose chambers had long-term retained bubbles, and wild-type animals which were transparent at the final movie frame were eliminated from the data analysis. Note that the average speed and frequency of scale-free time series obtained after linear transformations, such as differentiation and Fourier transformation, vary across scales of observation in accordance with the nature of scale-free time series (Fig. S1A and Supplemental Information). Scale-free analysis of time series obtained after linear transformations, in principle, reveals the same scale-free nature in the original time series.

### Statistical analysis

Student *t* tests and chi-squared tests for independence were performed with scipy.stats.chi2_contingency and scipy.stats.ttest_ind, respectively^98^.

## Data deposition

The *C. elegans* swimming activity time series and movie data reported in this paper have been deposited in Systems Science of Biological Dynamics (SSBD) database, https://ssbd.qbic.riken.jp/set/20190401/.

## Supplementary information

Supplementary Movie 1.

Supplementary Movie 2.

Supplementary Movie 3.

Supplementary Movie 4.

Supplementary Movie 5.

Supplementary Information.

Supplementary Figures.

Supplementary Figure and Movie Legends.

## Abbreviation

PKG: Cyclic GMP dependent kinase
