# Supplementary material for: C. elegans episodic swimming is driven by multifractal kinetics
Source: Sci Rep. 2020 Sep 8;10:14775. doi: 10.1038/s41598-020-70319-0 (PMC7478975; doi:10.1038/s41598-020-70319-0)
Supplement: Supplementary file 7 — Supplementary Figures. [file 41598_2020_70319_MOESM7_ESM.pdf]

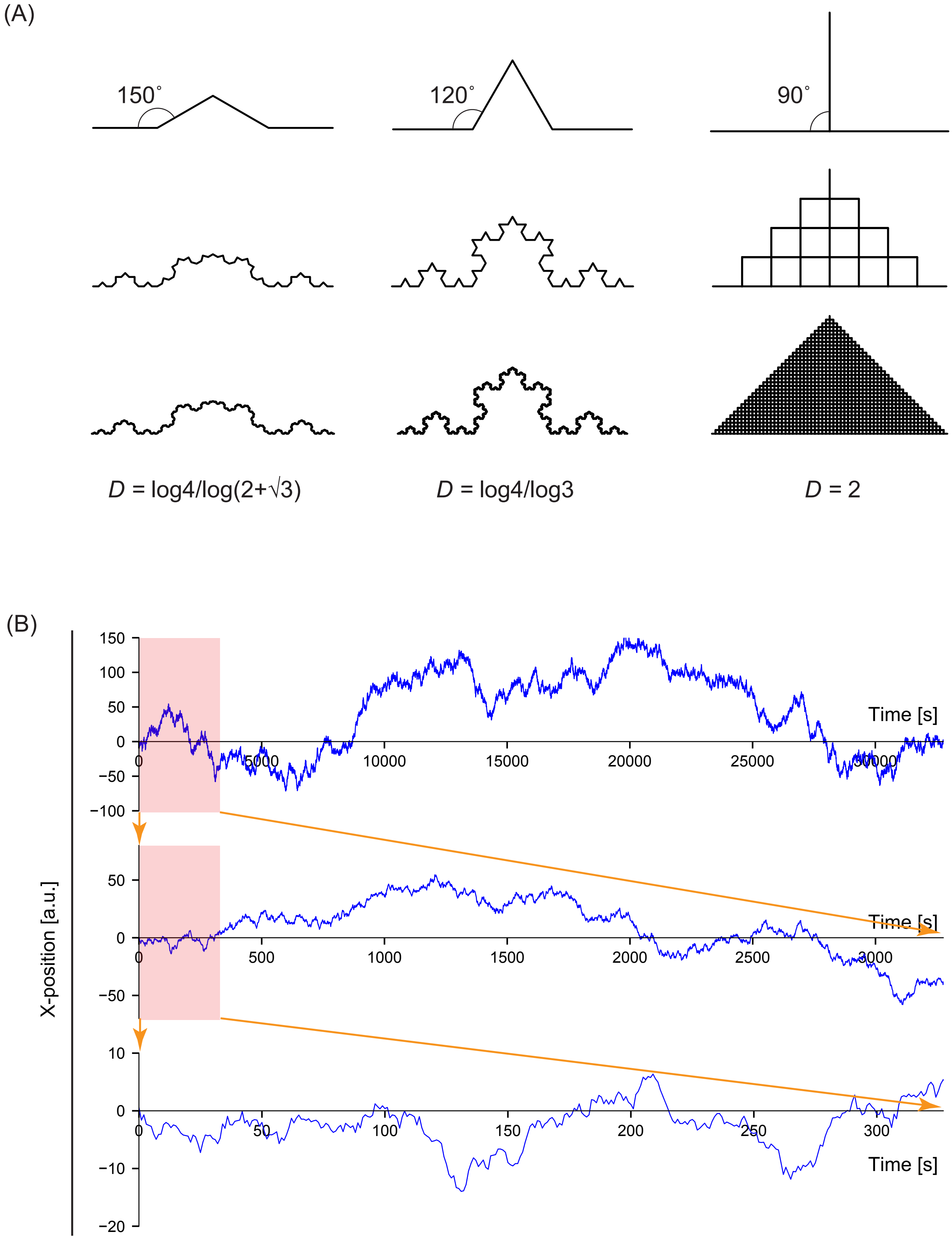

Figure S1 Ikeda et al

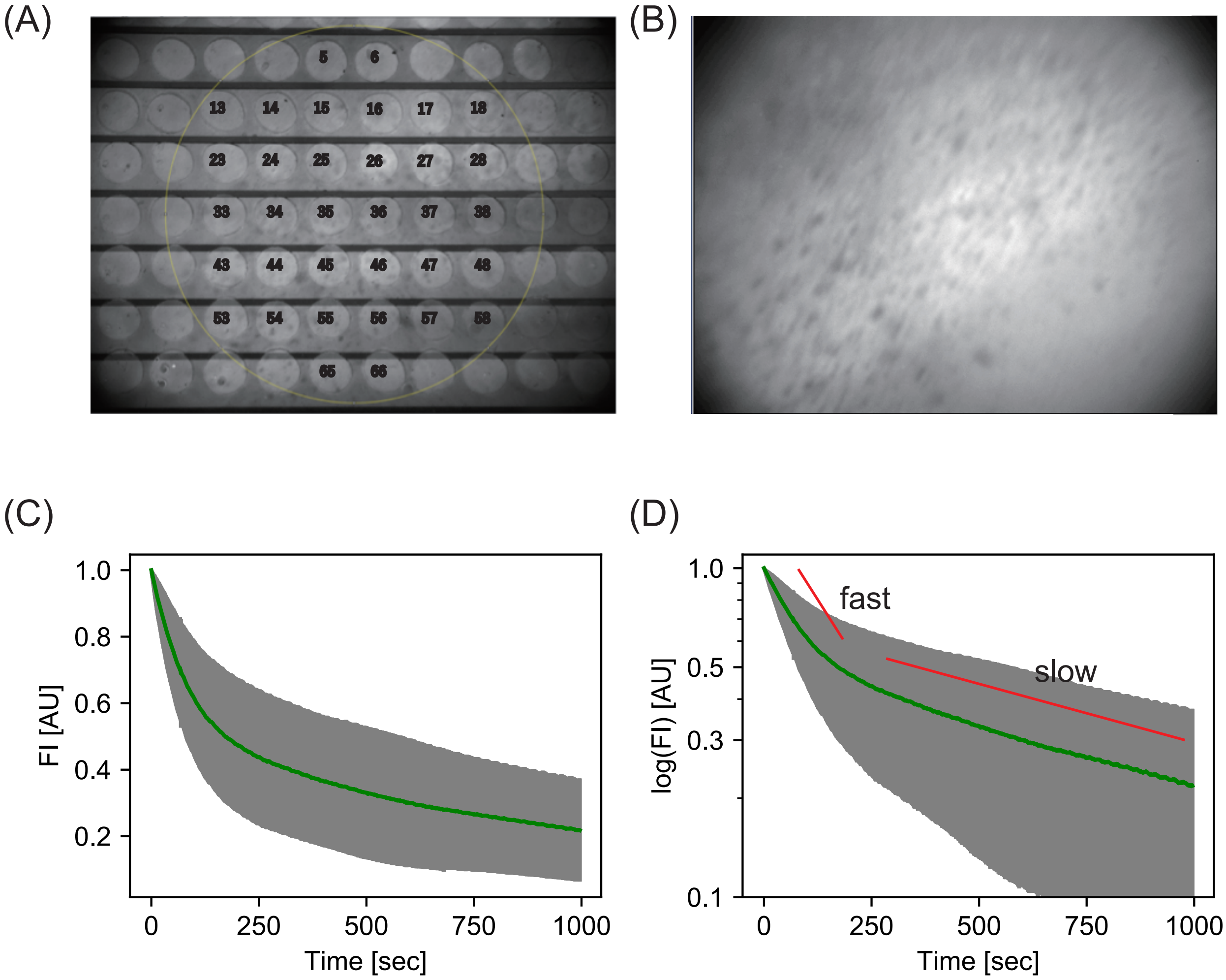

Figure S2 _IKeda et al

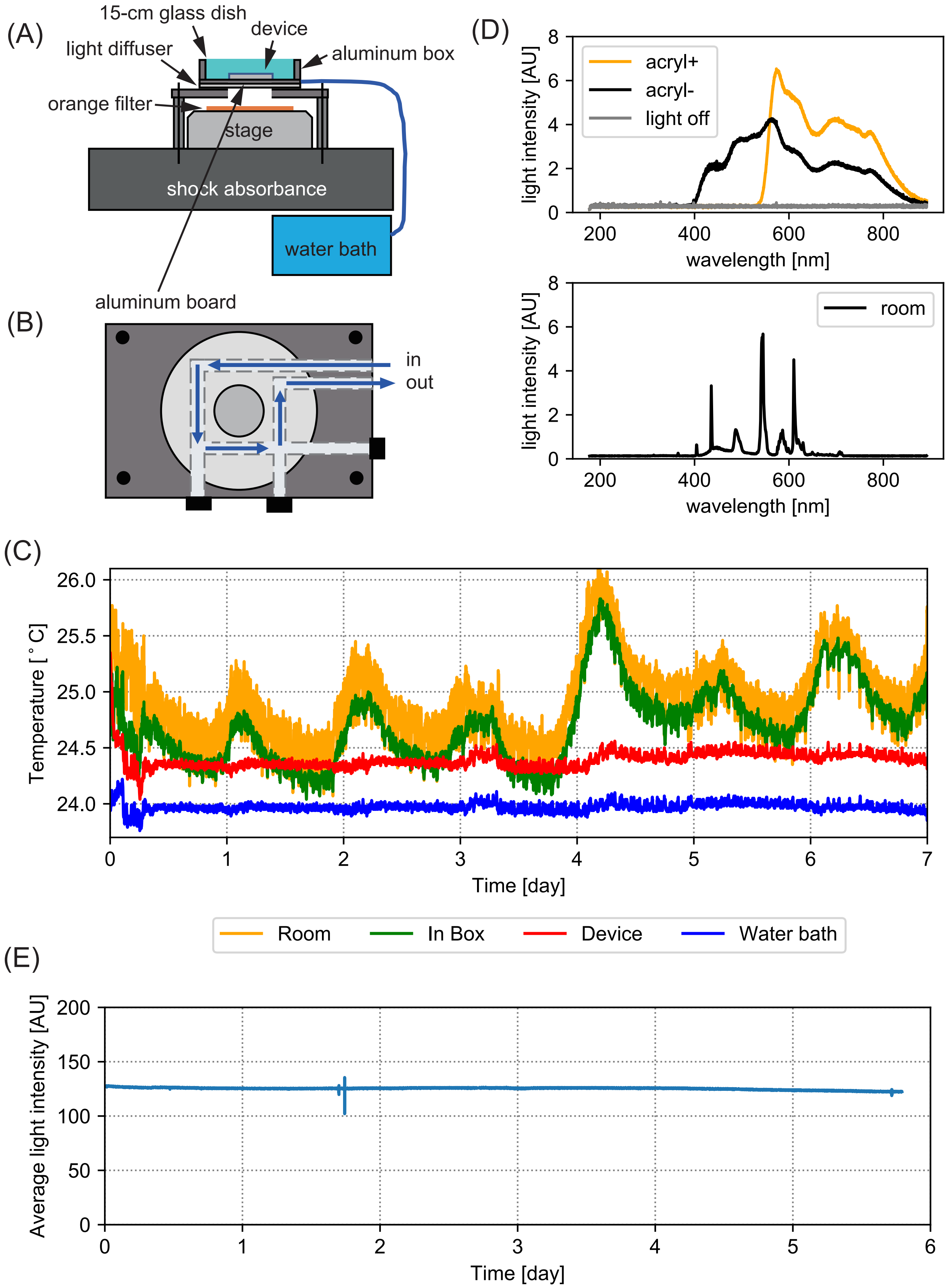

Figure S3_Ikeda et al

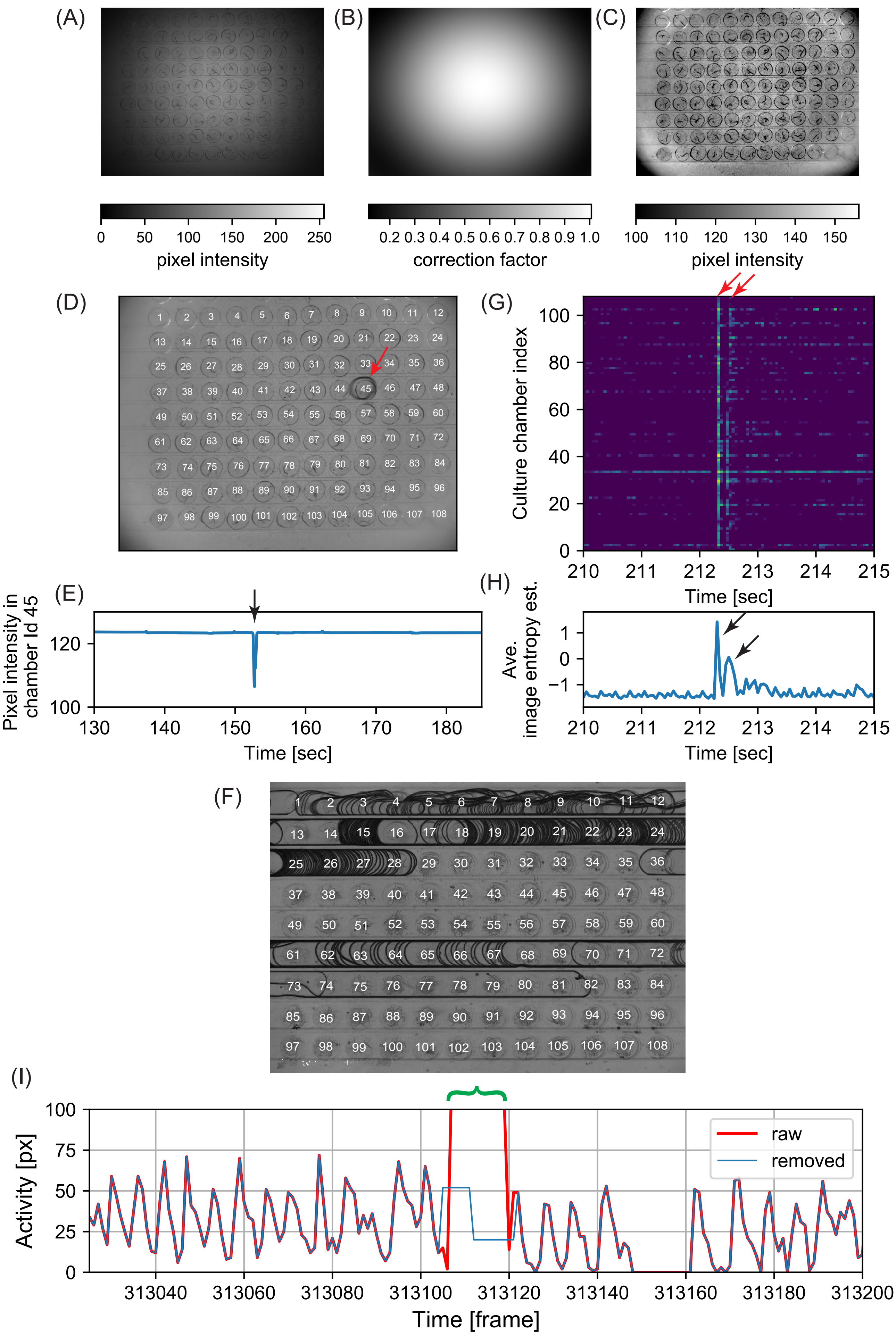

Figure S4_Ikeda et al

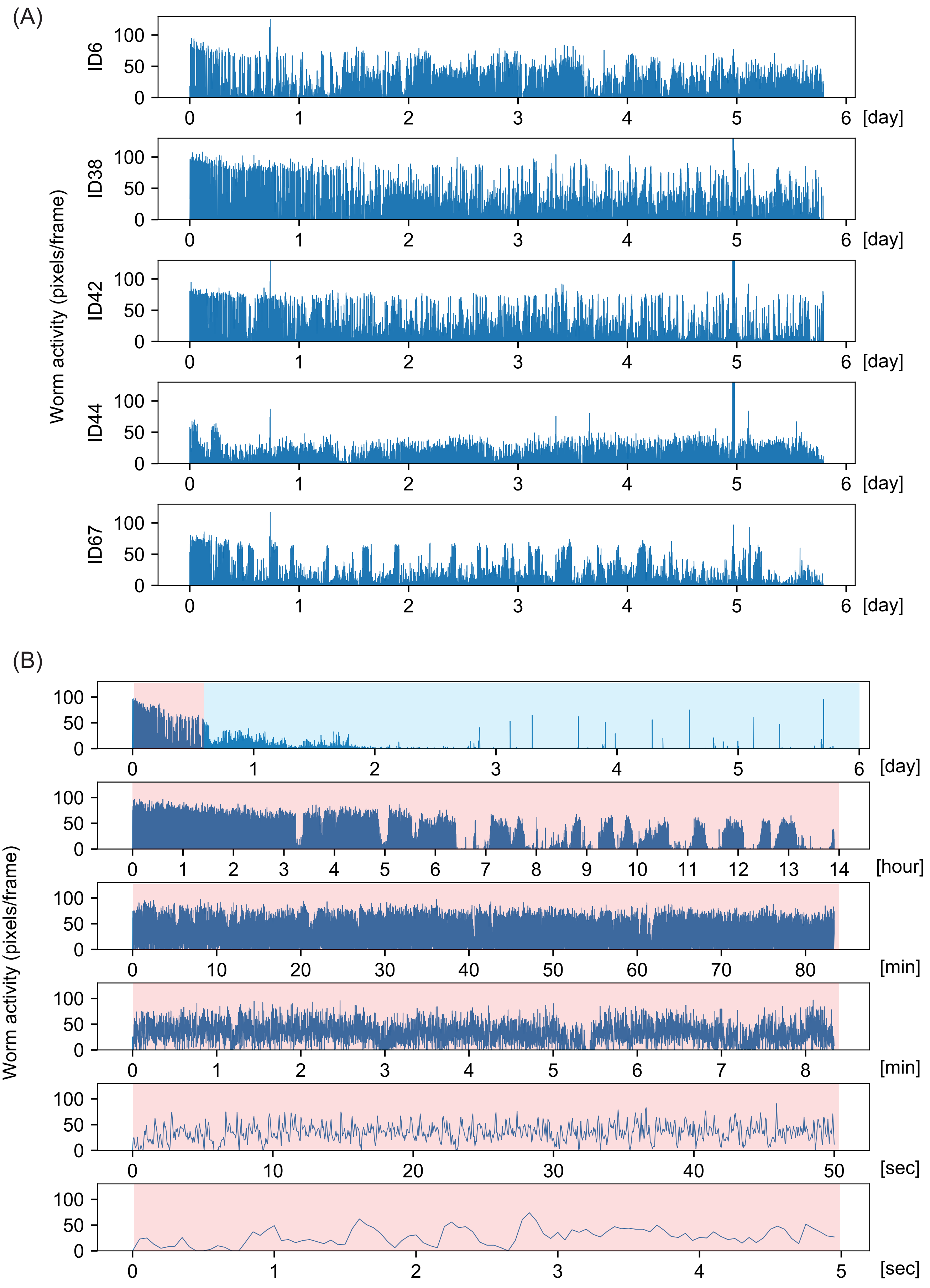

Figure S5_Ikeda et al

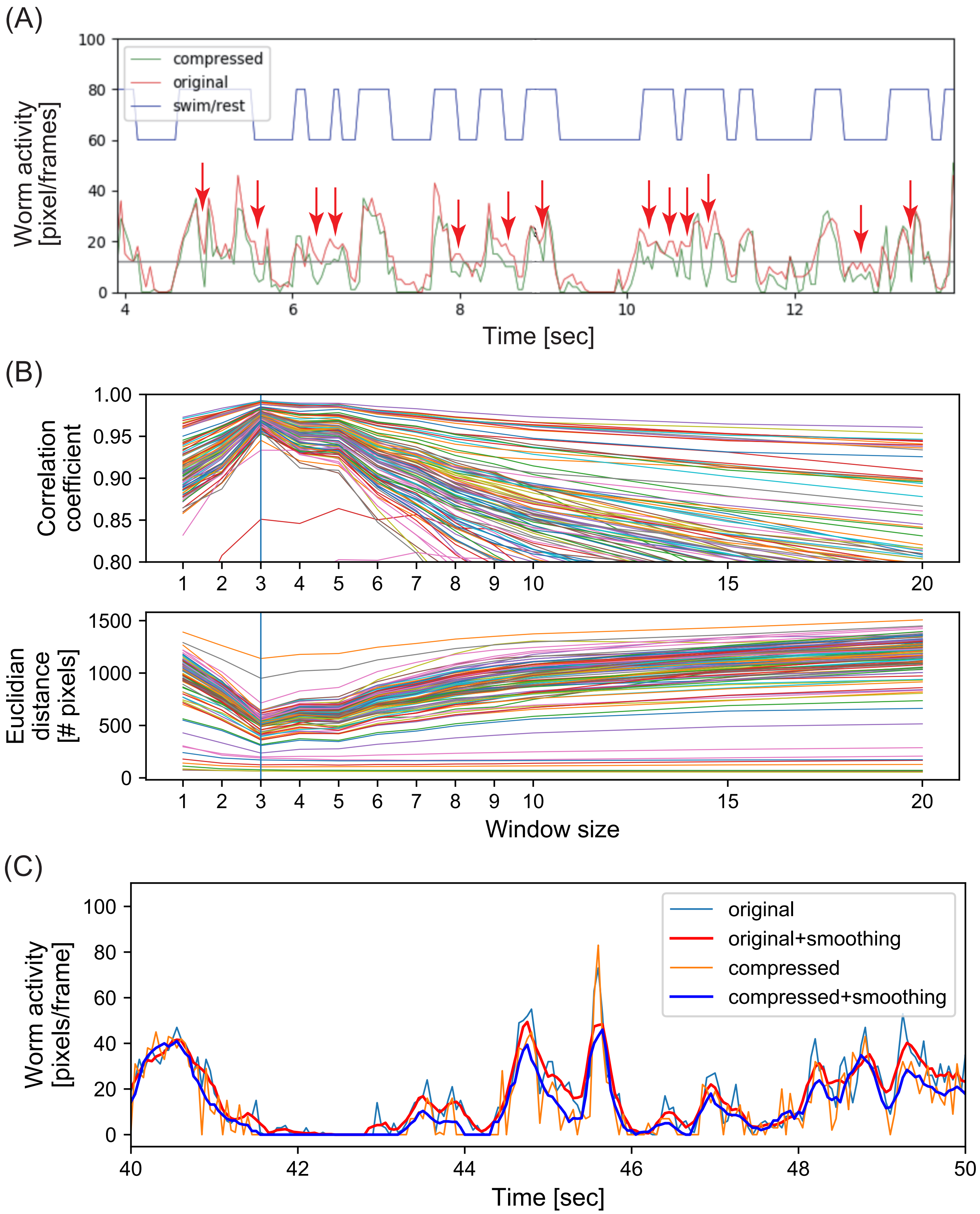

Figure S6 Ikeda et al
